# Medication-related quality of life among Ethiopian elderly patients with polypharmacy: A cross-sectional study in an Ethiopia university hospital

**DOI:** 10.1371/journal.pone.0214191

**Published:** 2019-03-28

**Authors:** Henok Getachew Tegegn, Daniel Asfaw Erku, Girum Sebsibe, Biruktawit Gizaw, Dawit Seifu, Masho Tigabe, Sewunet Admasu Belachew, Asnakew Achaw Ayele

**Affiliations:** Department of Clinical Pharmacy, University of Gondar, Gondar, Amhara, Ethiopia; Drake University College of Pharmacy and Heath Sciences, UNITED STATES

## Abstract

Polypharmacy among older patients has been associated with a decline in their quality of life. We aimed to assess the medication-related quality of life (MRQOL) among older patients with polypharmacy at Gondar University Hospital, Gondar, Ethiopia. A prospective cross-sectional study was carried out among 150 elder patients who had visited the internal medicine ward and ambulatory ward of Gondar referral hospital from March 25 to May 15, 2017, using a validated scale, Medication-Related Quality of Life Scale version 1.0 (MRQoLS-v1.0). A total of 150 older patients with polypharmacy participated in the study with a mean age of 70.06±5.12, andtwo-thirds of the participants (67.3%) were female. The overall prevalence of poor quality of life due to polypharmacy in the current study was found to be three fourth (75.3%) of the participants. Regarding the severity of impairment in MRQoL, Univariate analysis revealed that frequency of hospital visits (COR = 1.34, 95% CI, 1.02–1.77) and medication number (COR = 1.94, 95% CI, 1.33, 2.8) had a statistically significant positive association with the likelihood of having a severe impairment.The multivariate analysis also showed that one unit increase in the number of hospital visits (AOR = 1.45, 95% CI, 1.040–2.024) and medications greater than 5 (AOR = 1.91, 95% CI, 1.29, 2.84) increases 1.45 and 1.91 times the likely hood of posing severe impairment of MRQoL, respectively. As far as poor MRQoL quality of life is concerned, multivariate analysis did not show any significant association between the poor MRQoL;and Sociodemographic and clinical data of patients. The poor QoL associated with medication was very high in this study. Deprescribing should be sought by the health care providers to optimize drug therapy and minimize the polypharmacy related poor quality of life.

## Introduction

Polypharmacy, often defined as the use of five or more medications, has been linked with pervasive and negative impact both on patients and the health care system[1]. It has been associated with increased risk of drug-drug interactions, inappropriate dosing,andincreased overall health care costs as well as a variety of geriatric syndromes including functional decline[2]. Because of comorbidities, loss of functions and decreased autonomy associated with aging, older population are more prone to polypharmacy and poor health outcomesassociated with it. Many of these poor health outcomes are often subjective and challenging to measure. Patient-perceived quality of life (QoL), one of the poor health outcome associated with polypharmacy, has been widely used asa measure of patient care in clinical researchand health economic assessments[3]. The definition of polypharmacy has been a point of uncertainty. Ong GJ et al. study has determined that there has been an agreement among clinicians on the assessment of polypharmacy and its impact to cause harm to the patient,and other additional factors such as quality of prescription addition to the number of medication can contribute to patient health outcome in addition to the number of medications[4].

Health-related quality of life (HRQoL) has been defined as "the subjective perception influenced by the current health status of the ability to perform activities important for the person"[5, 6]. In response to the heightened importance of assessing HRQoL in examining the health status and analyze the effectiveness of health care interventions in older patients with polypharmacy[7], a range of instruments has been developed to measure HRQoL including the Medical Outcomes Study 36-item Short Form Health Survey (SF-36)[8, 9].

Recently, the role of medication as a determinant of patients’ overall health outcome has emerged as an issue demanding special attention [10]. QoL specific to medication burden is challengingtomeasure in older populations, taking into consideration the longitudinal changes in this outcome. Most of the studies that have examined the relationship between polypharmacy and QoL have done so from the perspective of adherence and used the generic health-related QoL (HRQoL), which may not have sufficient sensitivity to detect other medication-related factors and the transitory effect of medication change[8–14].

In the present study, we used the Medication-Related Quality of Life Scale version 1.0 (MRQoLS-v1.0) questionnaire, which is specific to patients with medication problems and has been recently validated as a measure of medication-related QoL (MRQoL) in patients with polypharmacy[15]. A better understanding of these medications related factors in older patients with polypharmacy may be useful to tailor interventions to the needs of individual patients in efforts to improve overall care and HRQoL.

## Materials and methods

### Study design and setting

An institutional-based quantitative cross-sectional survey was conducted on 150 older patients with polypharmacy who visited Gondar University Hospital (GUH) from March 1 to May 30, 2017, to assess MRQoL of older patients with polypharmacy. The hospital is located in Gondar town, northwest Ethiopia, 738km away from Addis Ababa and it the only referral and teaching center in the area to which majority of patients with chronic diseases including hypertension, diabetes mellitus, cancer,and asthma are referred.

### Sampling and recruitment strategies

A convenience sample of all older (≥ 65 years) patients who have been taking at least 5medications regardless of their diagnosis and who visited GUH from March 1 to May 30 for follow-up and medication refill were asked to participate. Patients who had severe physical or psychological problems which lead to the inability to complete the interview and those who refused to participatewere excluded from the survey. A total of 167 patients were approached during the study period; however, 13 patients were unwilling while 4 patients were unable to complete the questionnaire. Finally, 150 patients, who were willing and managed to complete the questionnaire with 89.8% response rate, were included in the study.

### Study instruments

We used the MRQoLS-v1.0questionnaire, which is specific to older patients with medication problems and has been recently validated as a measure of MRQoL among older patients with polypharmacy[15]. The tool has three domains: "Role limitations due to medication'' (6 items), ‘‘self-control'' (5 items), and ‘‘vitality'' (3 items) having a total of 14 items. Participants were asked to respond to every 14 items with six rated Likert scale response format (0 = none of the time, 1 = very rarely, 2 = rarely, 3 = occasionally, 4 = frequently, and 5 = all of the time) which are designed to assess QoL impaired due to use of multiple medications with an overall possible score from 0 to 70. As the different diseases vary in terms of their impact on health, each medical conditions should objectively be weighed to measure the comorbidity burden using the Charlson Comorbidity Index (CCI) [16].

Data were collected by three of the principal investigators through an interviewer-administered questionnaire. The questionnaire, first prepared in English, was translated to Amharic language and back to English to ensure that the translated version gives the proper meaning. It wasfurther pre-tested on 25 older patients with polypharmacy, who were not included in the final analysis, and appropriate modifications were done before the commencement of the actual study. The final questionnaire includes questions regarding the socio-demographic and treatment characteristics and a series of questions assessing MRQoL due to polypharmacy. The questionnaires areprovided in the supporting information section (S1 and S2 Quests).

### Statistical analysis

All the statistical analyses were done using Social Sciences (SPSS) software version 21.0 for Windows (SPSS Inc., Chicago, IL). As there was no published guideline for score classification, the authors used the following scoring classification for MRQoL based on the overall average score. The severity of impairment in QoL was rated as no/mild impairment (score from 0–23), moderate impairment (score from 24–46) and severe impairment (score from 47–70), while overall QoL was classified as good (score from 0–35) and poor (score from 36–70).

Frequencies and percentages were used to express different variables. Univariate and multivariable logistic regressions were used to come up with predictors of poor QoL. Variables with a significance level of less than 0.20 (*p* < 0.20) in the univariate analysis were included in the final model of multivariate logistic regression analysis. The results were adjusted for patients’ demographic and disease characteristics. Odds ratio (OR) with 95% CI were also computed along with corresponding *p*-value (*p*<0.05) as cut off points for determining statistical significance.

### Ethical considerations

This study was approved by the ethical committee of the University of Gondar with an approval number of SOP 913/2017. Written informed consent from the respondents was also obtained before conducting this study. Participants’ information obtained was kept confidential.

## Results

A total of 150 older patients with polypharmacy participated in the study with a response rate of 89.8% having a mean age of 70.1±5.1, ranging from 65 and 89 years. Nearly two-thirds of the participants (67.3%) was female. Majority of the participants 94 (62.7%) patients have visitedthehospital at least once before the time of enrollment (Table 1).

**Table 1:** Socio-demographic and clinical data of participants (N = 150).

*Variables*	*N*	*%*
***Age (Mean*** **±** ***SD)***	*70*.*1*±*5*.*1*	
***Age***		
*** 65–69***	*80*	*53*.*3*
*** 70–74***	*43*	*28*.*7*
*** 75–79***	*16*	*10*.*7*
*** ≥80***	*11*	*7*.*3*
***Sex***		
*** Male***	*49*	*32*.*7*
*** Female***	*101*	*67*.*3*
***Education***		
*** Cannot write and read***	*88*	*58*.*7*
*** Primary school***	*42*	*28*.*0*
*** Secondary school***	*16*	*10*.*7*
*** University/college***	*4*	*2*.*7*
***Setting***		
*** In patient***	*52*	*34*.*7*
*** Outpatient***	*98*	*65*.*3*
***Primary diagnosis***		
*** Hypertension***	*31*	*20*.*7*
*** Myocardial Infarction***	*3*	*2*.*0*
*** Chronic liver disease***	*6*	*4*.*0*
*** CAP***	*3*	*2*.*0*
*** Tuberculosis***	*2*	*1*.*3*
*** Congestive Heart Failure;***	*31*	*20*.*7*
*** Diabetic Mellitus***	*18*	*12*.*0*
*** Stroke***	*15*	*10*.*0*
*** Thyrotoxicosis***	*11*	*7*.*3*
*** Coronary artery disease***	*10*	*6*.*7*
*** Chronic Kidney Disease;***	*7*	*4*.*7*
*** Arthritis***	*5*	*3*.*3*
*** Asthma & COPD***	*6*	*4*.*0*
*** Others***	*2*	*1*.*3*
***N******o*** ***of prior hospital visits (Mean*** **±** ***SD)***	*1*.*1*±*1*.*4*	
***CCI (Mean*** **±** ***SD)***	*2*.*5*±*1*.*3*	
***N******o*** ***of medications (Mean*** **±** ***SD)***	*5*.*7* ±*1*.*2*	

Abbreviations: CAP: Community-acquired pneumonia; CCI: Charlson comorbidity index; COPD: Chronic obstructive pulmonary disease; SD: Standard deviation

Total of 831 medications was found, and the three most commonly prescribed medicationswere furosemide (74prescriptions), enalapril (72prescriptions) and aspirin (68prescriptions).

As shown in (Table 2), about 150 participants responded to 14 MRQoL questions having 3 domains. In the first domain of role limitations due to medication, a total of 64 patients (42.7%; 95%CI: 35%-51%) felt they took extra effort or had difficulty of performing the work or daily activities, yet, there had been a comparable percentage of participants (42%) who also thought they were limited in the work or other daily activities. Self-control domain showed that close to 63% (95%CI: 56%-71%) of the participants thought of themselves as a burden to others and 60.7% of the respondents also worried about disappointing others. Concerns about vitality factor;themajority of the participants (64.7%; 95% CI: 57%-72%) often have a reduced number of days feeling enthusiastic (Table 2).

**Table 2:** Patients’ response regarding the medication related quality of life due to polypharmacy.

*Items in each domain*	*None of the time N (%)*	*very rarely* *N (%)*	*Rarely* *N (%)*	*Occasionally* *N (%)*	*Frequently* *N (%)*	*All the time N (%)*	*Frequently and All the time % (95% CI)*
***A*. *Role limitations due to medication****–The domain mean score* ± *SD (range)2*.*9* ±*1*.*02*							
*1*. ***Cut down the amount of time you spent on work or daily activities***	*7(4*.*7)*	*24(16)*	*6(17*.*3)*	*42(28)*	*40(26*.*7)*	*11(7*.*3)*	*34*.*0%(26–42)*
*2*. ***Accomplish the work less than you would like***	*6 (4)*	*17 (11*.*3)*	*33(22)*	*46(30*.*7)*	*41(27*.*3)*	*7(4*.*7)*	*32*.*0% (24–40)*
*3*. ***Were limited in the work or other daily activities***	*2(1*.*3)*	*18 (12)*	*27 (18)*	*40 (26*.*7)*	*49(32*.*7)*	*14(9*.*3)*	*42*.*0% (34–50)*
*4*. ***Took extra effort or had difficulty performing the work or daily activities***	*6 (4)*	*12 (8)*	*29 (19*.*3)*	*39 (26)*	*51(34)*	*13 (8*.*7)*	*42*.*7% (35–51)*
*5*. ***Interfered with your social activities with family or friends***	*3 (2)*	*20 (13*.*3)*	*31 (20*.*7)*	*47 (31*.*3)*	*40 (26*.*7)*	*9 (6)*	*32*.*7% (25–40)*
*6*. ***Interfered with you recreational activities*, *such as exercise or watching TV***	*2 (1*.*3)*	*20 (13*.*3)*	*31 (20*,*7)*	*45 (30)*	*45 (30)*	*7 (4*.*7)*	*34*.*7% (27–42)*
***B*. *Self-control-*** *The domain mean score* ± *SD (range) 3*.*2*±*0*.*84*							
*7*. ***Felt frustrated or down-hearted***	*3 (2)*	*14 (9*.*3)*	*14 (9*.*3)*	*46 (30*.*7)*	*57 (38)*	*16 (10*.*7)*	*48*.*7% (41–57)*
*8*. ***Thought of yourself as a burden to others***	*5 (3*.*3)*	*7 (4*.*7)*	*16 (19*.*7)*	*27 (18)*	*56(37*.*3)*	*39 (26)*	*63*.*3% (56–71)*
*9*. ***Worried about disappointing others***	*2(1*.*3)*	*5(3*.*3)*	*17(11*.*3)*	*35(23*.*3)*	*47(31*.*3)*	*44(29*.*4)*	*60*.*7% (53–69)*
*10*. ***Had to cancel scheduled appointments or meetings***	*4 (2*.*7)*	*18 (12)*	*44 (29*.*3)*	*46 (30*.*7)*	*28 (18*.*7)*	*10 (6*.*7)*	*25*.*3% (18–32)*
*11*. ***Did not do work or other activities as a result of medication problems***	*3 (2)*	*16 (10*.*7)*	*35 (23*.*3)*	*60 (40)*	*29 (19*.*3)*	*7 (4*.*7)*	*24*.*0% (17–31)*
***C*. *Vitality*** *-The domain mean score+ SD (range)- 3*.*2*±*0*.*86*							
*12*. ***Had difficulty focusing on the task at hand or daily activities***	*4 (2*.*7)*	*8 (5*.*3)*	*39 (26)*	*51 (34)*	*44 (29*.*3)*	*4 (2*.*7)*	*32*.*0% (24–40)*
*13*. ***Had difficulty performing the work or daily activities as a result of feeling worn out***	*2 (1*.*3)*	*7 (4*.*7)*	*28 (18*.*7)*	*50 (33*.*3)*	*53 (35*.*3)*	*10 (6*.*7)*	*42*.*0% (34–50)*
*14*. ***Reduced the number of days feeling full of pep***	*-*	*13 (8*.*7)*	*10 (6*.*7)*	*30 (20)*	*78 (52)*	*19 (12*.*7)*	*64*.*7% (57–72)*

Note: During response for each question, 0 points for “none of the time” whereas 5 points for “All of the time” has been given. The median was the same for all 14 questions (i.e., Median = 3 or occasional). Confidence interval with 95% was computed to the merged response of frequently and all of the time response.

Of 150 participants, nearly half of patients (52.7%) had moderate impairment in MRQoLwhereas no/mild impairment and severe impairment was identified in 4.7% and 42.7% of participants, respectively. The overall prevalence of poor MRQoL due to polypharmacy in the current study was found to be three fourth (75.3%) of the participants (Table 3).

**Table 3:** Classification of impairment in MRQoL.

*Severity of impairment*	*Number*	*%*
*** ****None/mildimpairment(score0-23)*	*7*	*4*.*7*
*** ****Moderateimpairment(score24-46)*	*79*	*52*.*7*
*** ****Severeimpairment(score47-70)*	*64*	*42*.*7*
***Overall QoL*:***Good(score 0–35)*	*37*	*24*.*7*
*Poor (score 36–70)*	*113*	*75*.*3*

**Fig 1:**
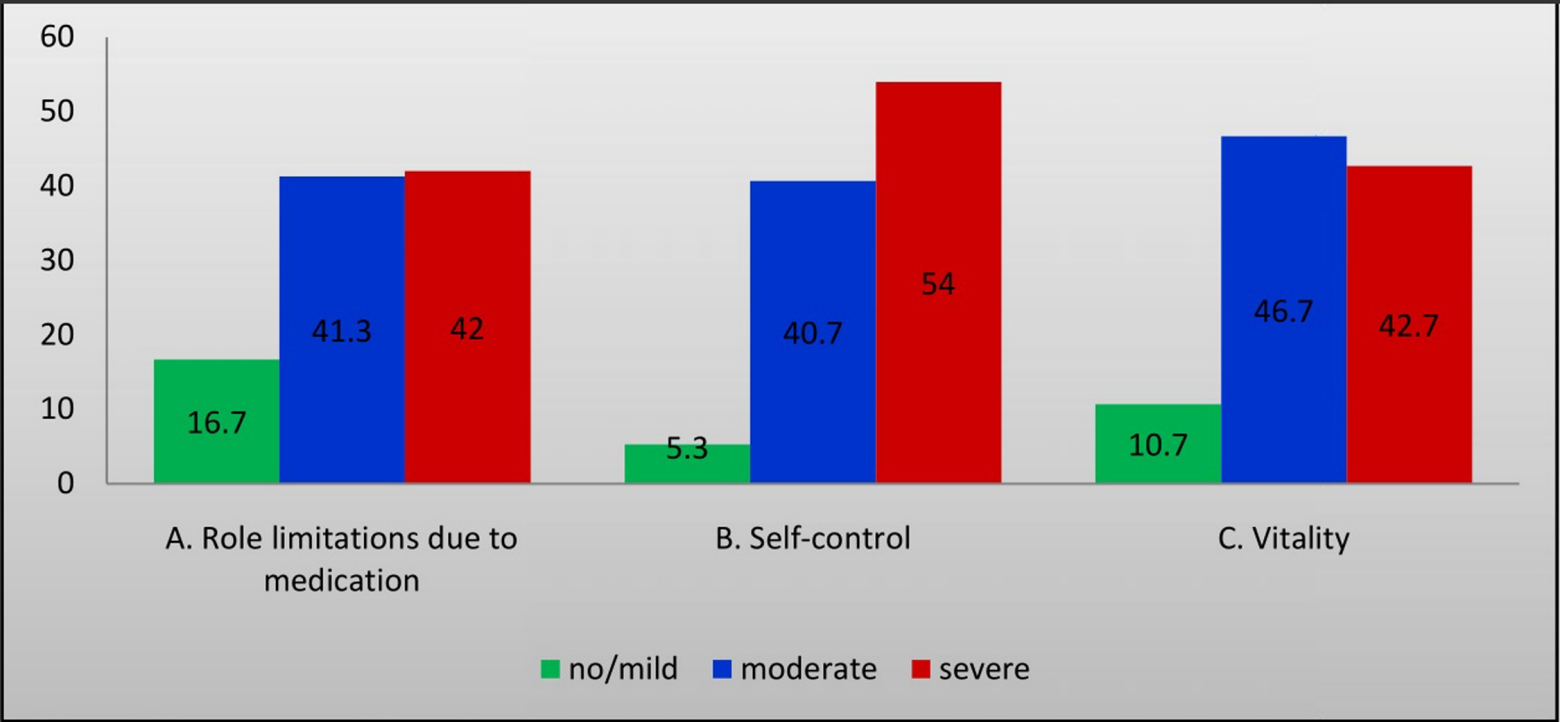
Percentage of the severity of impairment in each domain of medication-related quality of life (MRQoL). **A.** A domain with role limitations due to medications has6 questions with the severity of MRQoL (ranging from 0–30 score) and classified as no/mild (0–10), moderate (11–20), and severe (21–30). **B.** Self-control domain has 5 questions having MRQoL severity score ranging from 0–25 with subclassification of no/mild (0–8), moderate (9–16), and severe (17–25). **C.** Vitality domain also has3 questions with MRQoL severity score ranging 0–15 and classified as no/mild (0–5), moderate (6–10), and severe (11–15). All domain questions have a 6-point Likert scale ranging from 0 to 5.

The severity of impairment in QoL associated with polypharmacy has been determined in each domain of MRQoL. Higher proportion 81/150 (54%) of severe impairment in QoL was identified in self-control domain of MRQoL as shown in (Fig 1).

Regarding severity of impairment in MRQoL, Univariate analysis revealed that frequency of hospital visits (Crude odds ratio (COR) = 1.34, 95% Confidence Interval (CI), 1.02–1.77) and number of medications (COR = 1.93, 95% CI, 1.33, 2.8) had statistically significant positive association with the likelihood of having severe impairment of MRQoL.

The multivariate analysis also showed that one unit increase in the number of hospital visits (AOR = 1.45, 95% CI, 1.04–2.02) and medications greater than 5 (AOR = 1.91, 95% CI, 1.29, 2.84) increases 1.45 and 1.91 times the likely hood of posing severe impairment of MRQoL, respectively (Table 4).

**Table 4:** Relationship between predictive variables and the presence of a severe impairment in MRQoL.

*Variables*	*Severe impairment in MRQoL*		*OR(95%CI)*			
	*Yes*	*No*	*COR*	*p-value*	*AOR*	*p-value*
*** Age***						*0*.*15*
*** 65–69***	*28*	*52*	*0*.*94(0*.*25*,*3*.*49)*	*0*.*9*	*1*.*99(0*.*431*,*9*.*15)*	*0*.*38*
*** 70–74***	*25*	*18*	*2*.*43(0*.*62*, *9*.*56)*	*0*.*2*	*4*.*39(0*.*89*, *21*.*63)*	*0*.*07*
*** 75–79***	*7*	*9*	*1*.*36(0*.*28*, *6*.*58)*	*0*.*7*	*1*.*96(0*.*33*,*11*.*75)*	*0*.*46*
*** ≥80***	*4*	*7*	*1*	*-*	*1*	*-*
*** CCI (mean)***	*2*.*3*	*2*.*6*	*0*.*86(0*.*67*, *1*.*12)*	*0*.*27*	*0*.*75(0*.*56*,*1*.*01)*	*0*.*05*
***N******o*** ***of prior hospital visits (mean)***	*1*.*4*	*0*.*9*	***1*.*34(1*.*02*, *1*.*77)*** *	***0*.*04***	*1*.*45(1*.*04*,*2*.02)*	*0*.03*
*** N******o*** ***of medications (mean)***	*6*.*1*	*5*.*4*	*1*.*93(1*.*33*, *2*.8)**	*0*.001**	*1*.*91(1*.*29*, *2*.84)**	*0*.001**

Note: Severity of impairment in MRQoL has been classified as no/mild (score from 0–23), moderate (score from 24–46) and severe impairment (score from 47–70). However, only the presence of severe impairment has been analyzed in this table.

*P-value<0.05

**P-value<0.*001*

MRQoL has further been categorized into the poor and good overall QoL, and its risk factors have been analyzed. In univariate analysis, COR revealed that poor QoLis more likely to occur among patients prescribed with a higher number of medications (COR = 1.73, 95% CI, 1.03–2.88)(Table 5). After adjusting variables, multivariate logistic regression analysis showed that no significant association was found between the poor MRQoL and education, prior hospital visits and the number of medications as shown in (Table 5).

**Table 5:** Relationship between predictive variables and overall MRQoL.

*Variables*	*Overall MRQoL*		*OR(95%CI)*			
	*Poor*	*Good*	*COR*	*P-Value*	*AOR*	*P-Value*
***Education***						
*** Can’t write and read***	*63*	*25*	*1*	*-*	*1*	*-*
*** Primary school***	*35*	*7*	*1*.*98(0*.*78*, *5*.*05)*	*0*.*15*	*1*.*82 (0*.*700*,*4*.*708)*	*0*.*22*
*** Secondary school***	*14*	*2*	*2*.*78(0*.*59*, *13*.*12)*	*0*.*19*	*2*.*67(0*.*55*,*12*.*87)*	*0*.*22*
*** University/college***	*1*	*3*	*0*.*13(0*.*01*,*1*.*33)*	*0*.*08*	*0*.*16(0*.*015*,*1*.*634)*	*0*.*12*
***N******o*** ***of prior hospital visits (mean)***	*1*.*2*	*0*.*9*	*1*.*20(0*.*86*, *1*.*67)*	*0*.*27*	*1*.*07(0*.*760*,*1*.*502)*	*0*.*32*
***No of medications (mean)***	*5*.*8*	*5*.*3*	*1*.*73(1*.*03*, *2*.88)*	*0*.037*	*1*.*61 (0*.*959*,*2*.*695)*	*0*.*072*

Note: Overall QoL was classified as good (score from 0–35) and poor (score from 36–70). *P-value<0.05

## Discussion

In the current study, we enrolled 150 older patients with polypharmacy and assessed a self-reported QoL due to polypharmacy. To the best of our knowledge, there are no studies done to evaluate MRQoL. However, other studies [17, 18]employed the generic health-related QoL (HRQoL), which may not be adequate to measure the impact of medication change[2]. Moreover, studies [19–22] showed that the impact clinical pharmacist-led medication therapy management was not seen to improve HRQoL of older patients with polypharmacy even if such care improves other health outcomes such as minimizing drug-related problems. We have, therefore, employed MRQoL to measure QoL of older patients owing to medication which is more pronounced in the presence of polypharmacy.

Polypharmacy is linked with age, morbidity, and poor self-reported health[23].Studies done thus far assessed polypharmacy in people older than 65 and its related poor health outcome particularly HRQoL but did not measure its direct effect on QoL (MRQoL)[17, 18]. In this study, the majority of older patients with polypharmacy (75.3%) reported poor medication-related quality of life.

In the self-control dimension, most of the respondents (61%) claimed that they frequently or always worry about disappointing others. They (63.3%) also frequently or all of the time thought of themselves as a burden to others. As described by many patients this was because most of them have no source of income, and are supported by a family. As the number of medication increases, the cost also increases and some essential medications may not be available for which their caregivers are obliged to bring the medications from anywhere they get. Furthermore, the side effect management for the prescribed medications including dyspepsia and headache necessitates buying other medications leading to increased health cost.

In the vitality dimension, the majority of the participants (64.7%) stated that they had very few days with the feeling of full energy. On this point, most patients complain lack of hope by thinking that their medical condition is getting worse which has resulted in using many medications.

Regarding self-control dimension of MRQoL, problems related to anxiety-like disappointing others and feeling of a burden to others is pervasive among most participants in the current study. The research study by Alonso ML et al. also found that medication-related factors could affect the dimension of anxiety in Euro QoL 5D despite using a different tool (Euro QoL 5D)[18]. On the contrary, a feeling of anxiety symptoms such as worrying about disappointing others was found prevailing in the current study unlike a study done by Henderson JAet al.[8]. Loss of energy in daily activity is one of the typical sign of depression [24], which could be influenced by medication-related factors[18]. In the vitality dimension of this study has also reported a consistent finding that most patients felt they could spend very few numbers of days with full of energy.

From components of role limitations in our study questions; “Accomplish the work less than you would like” and “Were limited in the work or other daily activities,” was defined as a frailty in another study which tried to assess the cut points of polypharmacy and outcomes. “Took extra effort or had difficulty performing the work or daily activities” also defined as a disability in the same study and our results were mostly consistent with it[18]. In addition to this, the outcome regarding role limitation was also comparable with the result of other study done by Rosso ALet al. [25]which took polypharmacy as geriatric syndromes and it resulted in disability in older women. The components of role limitations discussed above were assessed in another study[26]as functional ability and consistent with our results. In another study, most components of role limitations were described as a physical component summary that is associated with daily living activities, and polypharmacy could impair the daily living activities [8].

The result of our study on impairment of QoL due to multiple medications use was consistent with the research done by Hajjar ER et al. which assessed the effects of increased medication use on the geriatric syndrome [27]. In our study, this denotes the cumulative effect of role limitation, vitality and self-control problems that in turn possess problem on MRQoL. Polypharmacy was also associated with poor QoLunlike the study done by Lalic Set al.[28]. This deviated result may be due to the difference in settings where the researches have been conducted.

In this study, results for the influence of age, sex, educational status and comorbidities on multi-medication use and MRQoL were not conclusive; this can be explained by a small number of participants used and high prevalence of chronic medical conditions. The associations between education, age, sex comorbid conditions, and educational status need further investigation, and also other social and environmental factors need to be seen. This finding on age and sex were similar to the result of a previous study [8] done in South Dakota assessing HRQoL on a sample of 63 patients.

In the more conservative classification of MRQoL, the increased number of hospital visits and medications greater than 5 remains significantly associated with severe impairment of MRQoL even after controlling for age and CCI in multivariate analysis. This is in agreement with other studies [18, 29, 30]done elsewhere that showed patients with a higher number of medications reported the worst results in a quality of life.

MRQoL was also classifiedinto two as poor and good,and no independent factors were identified to have a significant association with poor MRQoL even though higher number medications tend to pose poor MRQoL in univariate analysis. In contrast, other studies [29–35]that assessed HRQoL in people older than 65 years, and poorer HRQoL was associated with age, female sex, functional impairment, depression, chronic diseases or polypharmacy. The severity of the illness itself and other extrinsic factors might have contributed to poorer HRQoL which in return leading to such discrepancy with MRQoL of our study.

As an effort to improve medication-related QoL in older patients, various researchers recommend deprescribing of inappropriate polypharmacy to optimize medication use, thereby reducing inappropriate prescribing and improving health outcomes [36–40]. Any attempt to discontinue inappropriate prescriptions necessitates the active participation of patients in the decision-making process. A study done in the same setting gave a promising result in that many older patients could be willing to discontinue their medications if their doctor decides to deprescribe [40] which enable to minimize the burden of medications leading to a better QoL.

The study has suffered some limitations. Cross-cultural validity, reliability, and psychometric property of the Amharic version of MRQoL have not been done. The cutoff point for the MRQoL score was not determined by testing it against other standard measures using sensitivity/specificity data. The amount of time of exposure to polypharmacy was not analyzed which may affect the QoL, due to more extended use of polypharmacy. In addition to this, this research does not investigate the effects that different type of medications, medication adherence, and status of medication-related problems could have on MRQoL.

## Conclusion

The overall prevalence of poor MRQoL was 75.3% that implies polypharmacy results in poor QoLinolder patients. The frequency of hospital visit and medication number was the independent predictors for severe impairment in MRQoL. This study needs further investigation to see the correlation between independent variables and dependent outcomes. Physicians should seek Deprescribing aiming at minimizing inappropriate polypharmacy in older patients.

## Supporting information

S1 Data This is the study's underlying data set. (ZIP)

S1 Quest This is the English version of the questionnaire. (DOCX)

S2 Quest This is the Amharic version the questionnaire. (DOCX)
