# Supplementary material for: Medication-related quality of life among Ethiopian elderly patients with polypharmacy: A cross-sectional study in an Ethiopia university hospital
Source: PLoS One. 2019 Mar 28;14(3):e0214191. doi: 10.1371/journal.pone.0214191 (PMC6438590; doi:10.1371/journal.pone.0214191)
Supplement: S1 Quest — (DOCX) [file pone.0214191.s002.docx]

Medication-related quality of life among elderly people with polypharmacy in Gondar University Hospital using MRQoLS-v1.0: A cross sectional study

**Section I demographics data and clinical data**

1. Age____________
2. Sex: Male Female
3. Educational level
4. Can’t write and read
5. Primary school
6. Secondary school
7. University/college
8. Reason of hospital visit:__________________
9. Co-morbidities:

|  |
| --- |

1. CCI_________________________
2. Number of visits of the hospital (In-patient/out-patient) in the past year___________
3. Medications prescribed

| Medication | STRENGTH | ROA | FREQUENCY | DURATION | START DATE |
| --- | --- | --- | --- | --- | --- |
|  |  |  |  |  |  |

**Section II. Medication-related quality of life**

The following questions will reflect the quality of life because of taking multiple medications. The response include A six-point Likert scale where 1 =none of the time, 2=very rarely, 3= rarely, 4=occasionally, 5= frequently, 6= all of the time.

| Domains | Questions | Response | | | | | |
| --- | --- | --- | --- | --- | --- | --- | --- |
|  |  | 1 | 2 | 3 | 4 | 5 | 6 |
| 1. Role limitations due to medication | 1. Cut down the amount of time you spent on work or daily activities |  |  |  |  |  |  |
|  | 1. Accomplish the work less than you would like |  |  |  |  |  |  |
|  | 1. Were limited in the work or other daily activities |  |  |  |  |  |  |
|  | 1. Took extra effort or had difficulty performing the work or daily activities |  |  |  |  |  |  |
|  | 1. Interfered with your social activities with family or friends |  |  |  |  |  |  |
|  | 1. Interfered with you recreational activities, such as exercise or watching TV |  |  |  |  |  |  |
| 1. Self-control | 1. Felt frustrated or downhearted |  |  |  |  |  |  |
|  | 1. Thought of yourself as a burden to others |  |  |  |  |  |  |
|  | 1. Worried about disappointing others |  |  |  |  |  |  |
|  | 1. Had to cancel scheduled appointments or meetings |  |  |  |  |  |  |
|  | 1. Didn’t do work or other activities as a result of medication problems |  |  |  |  |  |  |
| 1. Vitality | 1. Had difficulty focusing on the task at hand or daily activities |  |  |  |  |  |  |
|  | 1. Had difficulty performing the work or daily activities as a result of feeling worn out |  |  |  |  |  |  |
|  | 1. Reduced the number of days feeling full of pep |  |  |  |  |  |  |
