# Supplementary material for: Medication-related quality of life among Ethiopian elderly patients with polypharmacy: A cross-sectional study in an Ethiopia university hospital
Source: PLoS One. 2019 Mar 28;14(3):e0214191. doi: 10.1371/journal.pone.0214191 (PMC6438590; doi:10.1371/journal.pone.0214191)
Supplement: S2 Quest — (DOCX) [file pone.0214191.s003.docx]

ክፍል ሁለት -ከመድሃኒት ጋር የተገናገኘ የኑሮ ጥራት

የሚከተሉት ጥያቄዎች ብዙ መድሃኒት በሚወስዱ ታካሚዎች ዘንድ ያለ የኑሮን ጥራት ለመለካት የምንጠቀምባቸው መመዘኛዎች ናቸው፡፡ መልሶቹን እንደሚከተለው መመለስ ይችላሉ፤

1. በጭራሽ 4. አልፎ አልፎ
2. በጣም ያልተለመደ 5. በተደጋጋሚ
3. ያልተለመደ 6. ሁል ጊዜ

| ዝርዝር | ጥያቄዎች | መልስ | | | | | |
| --- | --- | --- | --- | --- | --- | --- | --- |
|  |  | 1 | 2 | 3 | 4 | 5 | 6 |
| ሀ. በመድሃኒቱ ምክንያት የተገደቡ ተግባራት | 1.ከሚሰሩበት ወይም የእለት ተለት ተግባሮን ከሚያከናውኑበት ሰአት ላይ መቀነስ |  |  |  |  |  |  |
|  | 2.ስራዎትን ካቀዱት በታች ማከናወን |  |  |  |  |  |  |
|  | 3.በስራዎት ወይም በእለት ተለት ተግባሮት ላይ የተገደነ እንቅስቃሴ ማድረግ |  |  |  |  |  |  |
|  | 4.ስራዎትን ወይም የእለት ተለት ተግባሮትን ለማከናወን እገዛ መፈለግ |  |  |  |  |  |  |
|  | 5.ከማህበራዊ ህይወት፣ ከቤተሰብ እና ከጓደኛዎ ጋር ባለዎት ግንኙነት መሰናክል መሆን |  |  |  |  |  |  |
|  | 6.በመዝናኛ ህይወቶ ላይ መሰናክል መሆን ( ለምሳሌ ፤ ቴሌቪዝን መመልከት፣እንቅስቃሴ ማድረግ እና የመሣሠሉትን...) |  |  |  |  |  |  |
| ለ. ራስን መገደብ | 7. ተስፋ የመቁረጥ ወይም የበታችነት ስሜት መሰማት |  |  |  |  |  |  |
|  | 8. ራስን እንደ ሸክም አድርጎ መቁጠር |  |  |  |  |  |  |
|  | 9. ሌሎችን አስቸግሪያለሁ ወይም ቅር አሰኝቻለሁ ብሎ መጨነቅ |  |  |  |  |  |  |
|  | 10. ታቅደው የነበሩ ቀጠሮዎችን ወይም ስብሰባዎችን መሰረዝ |  |  |  |  |  |  |
|  | 11. በመድሀኒቱ ምክንያት ስራን ወይም ሌሎች ተግባራትን ማድረግ አለመቻል |  |  |  |  |  |  |
| ሐ. ጥንካሬ | 12. በስራዎት ወይም በእለት ተለት ተግባሮት ላይ ትኩረት ማድረግ አለመቻል |  |  |  |  |  |  |
|  | 13. አልቆልኛል ወይም አብቅቶልኛል ብሎ በማሰብ ስራዎትን ወይም ሌሎች ተግባራትን ማከናወን አለመቻል |  |  |  |  |  |  |
|  | 14. በደስታ የሚያሳልፏቸውን ቀናት መቀነስ |  |  |  |  |  |  |
|  |  | | | | | | |
